# India's JSY cash transfer program for maternal health: Who participates and who doesn't - a report from Ujjain district

**DOI:** 10.1186/1742-4755-9-2

**Published:** 2012-01-24

**Authors:** Kristi Sidney, Vishal Diwan, Ziad El-Khatib, Ayesha de Costa

**Affiliations:** 1 Division of Global Health, Nobels Väg 9, Karolinska Insitutet, Stockholm, Sweden; 2 Department of Public Health and Environment, RD Gardi Medical College, Agar Road, Surasa, Ujjain, Madhya Pradesh, India

**Keywords:** Janani Suraksha Yojana (JSY), Conditional Cash Transfer (CCT), Demand-Side Financing, India

## Abstract

**Background:**

India launched a national conditional cash transfer program, Janani Suraksha Yojana (JSY), aimed at reducing maternal mortality by promoting institutional delivery in 2005. It provides a cash incentive to women who give birth in public health facilities. This paper studies the extent of program uptake, reasons for participation/non participation, factors associated with non uptake of the program, and the role played by a program volunteer, accredited social health activist (ASHA), among mothers in Ujjain district in Madhya Pradesh, India.

**Methods:**

A cross-sectional study was conducted from January to May 2011 among women giving birth in 30 villages in Ujjain district. A semi-structured questionnaire was administered to 418 women who delivered in 2009. Socio-demographic and pregnancy related characteristics, role of the ASHA during delivery, receipt of the incentive, and reasons for place of delivery were collected. Multinomial regression analysis was used to identify predictors for the outcome variables; program delivery, private facility delivery, or a home delivery.

**Results:**

The majority of deliveries (318/418; 76%) took place within the JSY program; 81% of all mothers below poverty line delivered in the program. Ninety percent of the women had prior knowledge of the program. Most program mothers reported receiving the cash incentive within two weeks of delivery. The ASHA's influence on the mother's decision on where to deliver appeared limited. Women who were uneducated, multiparious or lacked prior knowledge of the JSY program were significantly more likely to deliver at home.

**Conclusion:**

In this study, a large proportion of women delivered under the program. Most mothers reporting timely receipt of the cash transfer. Nevertheless, there is still a subset of mothers delivering at home, who do not or cannot access emergency obstetric care under the program and remain at risk of maternal death.

## Background

The use of maternal health care services remains low throughout most South Asian countries despite continued efforts to strengthen the infrastructure, drug supply and human resource capabilities [1]. While these improvements are important to deliver services, they do not address many of the access barriers faced by the poor [2]. Demand-side financing initiatives are specifically intended to reduce cost related access barriers for vulnerable groups by giving them purchasing power to use a designated service [3]. The concept involves funneling government or donor funds directly to a selected group. There are various approaches, one of them being a conditional cash transfer (CCT). A traditional CCT bestows a financial incentive directly to the beneficiary if the recipient complies with a certain set of prerequisites [4,5].

Varying degrees of success have been reported from similar CCT programs in South Asia; Nepal, India, and Bangladesh. All of the programs have experienced increased utilization of maternal health care services [6-8], however barriers reported from Bangladesh [8,9] and Nepal [10] include issues pertaining to the timely reimbursement of the cash incentive for beneficiaries and providers and difficulties for the most poor women to gain access to the programs.

Beginning in 2005, India launched a national CCT program to promote institutional delivery, Janani Suraksha Yojana (JSY or 'Safe Motherhood Program') [11]. The JSY program is fully funded by the Government of India and operates under the National Rural Health Mission (NRHM). The program has attracted considerable interest across the globe due to its size, scope and investment received [12]. Functional nationwide, it is the largest cash transfer program in the world [7]. In 2008-2009, $275 million was allocated to the program and 8.43 million women benefited from it, representing nearly a third of all women who deliver in the country annually [12]. The eligibility criteria for the program differs depending on the province. Women delivering in non-high focus provinces (provinces with a relatively better in-facility birth proportion) are only eligible for the cash benefit for their first two live births, and if they have a government issued below the poverty line card or belong to a scheduled caste or tribe. The program deviates from the traditional CCT model in high focus provinces, those with a low in-facility birth proportion, as it does not include a conditionality component. All women who deliver in a public facility receive the cash incentive. In Madhya Pradesh, a high focus province, rural women receive $28 (1400 INR) whereas urban women receive $20 (1000 INR) upon delivery in a public facility. All services provided in the public health sector are free of charge to the end user. The program is supported in the community through the selection of an accredited social health activist (ASHA). The ASHA is a female resident of the village who is incentivized to motivate women to deliver at public facilities under the program [13].

To date, there have been few research reports on this large scale demand-side financing program for maternal health. Previous assessments have been descriptive [14], process oriented in selected states [15], or based on secondary data collection [7]. Little has been documented on factors that influence how beneficiaries and non-beneficiaries interact with the services provided. This paper studies the extent of program uptake, reasons for participation/non participation and factors associated with not participating in the cash transfer program in one district in India. It also studies the timeliness of receipt of the cash incentive by mothers and the role of the ASHA in the delivery.

## Methods

### Study Site

A community based cross-sectional study was performed in Ujjain district, one of the 50 administrative districts in the province of Madhya Pradesh, India between January and May 2011. Ujjain district has a population of 1.9 million people, 61% of whom are rural and 25% of whom belong to scheduled caste [16]. Scheduled castes, backward castes and scheduled tribes are a group of people who were historically subject to social disadvantage and exclusion. They are awarded special status by the Constitution of India (listed in a schedule) and thus recipients of specific social benefits [17]. Ujjain also has a literacy rate of 73% [18] and an infant mortality rate of 59/1 000 live births [19].

**Figure 1:**
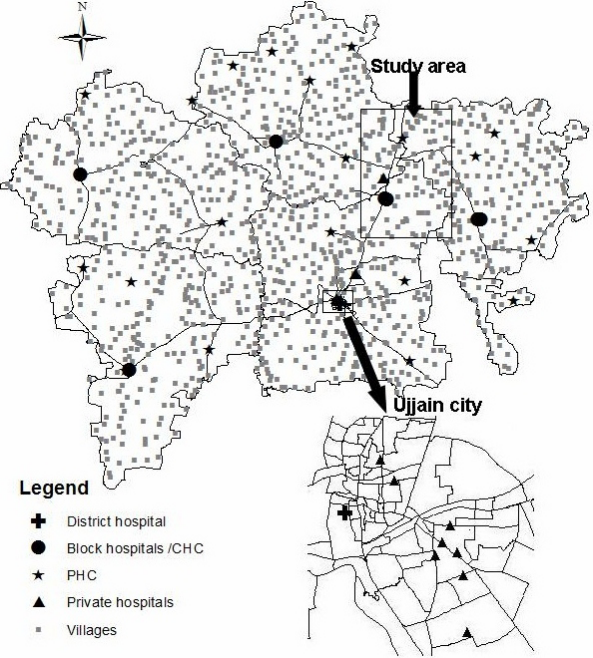
**Map of Study Site, Ujjain District and Facilities Visited by the Women**.

This study was performed in an epidemiological field study area that is under the routine surveillance of the Department of Community Medicine, Medical College Ujjain. The field area consisted of 60 villages from three different community development blocks in Ujjain district (figure 1). Records on all births and deaths in these villages are routinely maintained. This information was made available to the research team and served as the starting point for the study. Access to the data from the epidemiological field study area and the location of the medical university in Ujjain district were reasons why it was selected for the study. Thirty villages were randomly selected from the field area, ten from each block.

The health infrastructure in the study area is similar to the health system in India, a mix of public and private providers. The public sector in rural areas consist of a three-tier structure; (i) at the lowest level, a sub-center run by a female health worker; (ii) at the intermediary level, a Primary Health Center (PHC) with a medical officer and other paramedical staff; and (iii) at the higher level, a Community Health Center (CHC) with obstetric specialists and inpatient beds. Tertiary care is provided by the district hospital. PHCs, CHCs and private facilities relevant to our study are shown in figure 1. The private facilities shown on the map represent most of the private facilities that the study women visited. There are many other private hospitals in the district which are not indicated.

### Study Participants

Eligible participants included women residing in the villages who gave birth in 2009. The database maintained and updated at the medical college was used to identify the women. Trained research assistants visited these women at home and invited them to participate. Of the 742 eligible mothers, all 418 women that were home at the time of the visit gave witnessed verbal consent to participate.

### Data Collection

A semi-structured, pilot tested questionnaire was designed to elicit information on socio-demographic characteristics and history of previous pregnancies. Knowledge of and participation in the JSY program was also ascertained. With regard to the most recent pregnancy, information on the place of delivery, delivery type, antenatal care (ANC) visits, role of ASHA in facilitating the delivery, infant and maternal outcomes were collected. Information on receipt of the cash incentive by the mother was also included.

The interview was conducted in Hindi with the mother in her home. If the mother was unavailable at the time of interview, reasons for her absence were noted. If the mother was deceased, close relatives (mother in law/husband) were interviewed.

### Dependent variables

A categorical dependent variable was created based on the place of delivery; a 'JSY facility' (program delivery), 'private facility' or 'at home'.

### Independent variables

Socio-demographic variables: The independent variables included in the analysis were mother's age, poverty status determined by the presence of a below the poverty line (BPL) card, education, and caste (disadvantaged groups referred to in the study included other backward castes, scheduled castes and scheduled tribes).

Pregnancy related variables: Prior knowledge of the JSY program (this was ascertained if mothers knew they were entitled to a cash transfer when they delivered in a public facility), parity, ANC visits, transportation time to the facility, type of delivery, and maternal and neonate outcome.

### Data management and Analysis

Data was entered in EpiData (version 3.1) then transferred to STATA 10 for the analysis. Basic descriptive statistics as well as tests of significance for proportions were computed. To identify predictors for the outcome variable, a multinomial regression analysis was used. The independent variables found to be significant in a bivariate level analysis were then included in a regression model. Odds ratio (OR) 95%CI; were presented.

## Ethical Considerations

Ethical approval was granted by the Ethics Committee of R.D. Gardi Medical College, Ujjain.

## Results

### Background characteristics and program uptake (Table 1)

**Table 1:** Characteristics of women who gave birth in Ujjain District in 2009 by place of delivery

		N (%)	JSY N (%)	Private N (%)	Home N (%)	p value
**(a) Study population characteristics**						
Age						
	≤25 years	306 (73)	233 (73)	41 (76)	32 (70)	
	>25 years	112 (27)	85 (27)	13 (24)	14 (30)	0.77
Poverty level						
	APL	204 (49)	143 (45)	37 (69)	24 (52)	
	BPL	214 (51)	175 (55)	17 (31)	22 (48)	**0.01**
Education						
	Primary, high school or higher	220 (53)	166 (52)	39 (72)	15 (33)	
	No formal education	198 (47)	152 (48)	15 (28)	31 (67)	**<0.01**
Caste						
	Disadvantaged groups	118 (28)	79 (25)	28 (52)	11 (24)	
	General	300 (72)	239 (75)	26 (48)	35 (76)	**<0.01**
**(b) Pregnancy Related**						
Knowledge of JSY	Yes	373 (89)	304 (96)	37 (68)	32 (70)	
	No	45 (11)	14 (4)	17 (32)	14 (30)	**<0.01**
Number of previous deliveries						
	1 delivery	143 (34)	115 (36)	21 (39)	7 (15)	
	≥2 deliveries	275 (66)	203 (64)	33 (61)	39 (85)	**0.02**
Number of ANC visits attended						
	0-3 visits	221 (53)	179 (56)	10 (19)	32 (70)	
	≥4 visits	197 (47)	139 (44)	44 (81)	14 (30)	**<0.01**
Time spent to travel to the place of delivery (minutes)						
	<median 45 minutes	206 (49)	189 (60)	17 (29)	0	**<0.01**
	>median 45 minutes	212 (51)	125 (40)	41 (71)	46(100)	
Type of delivery						
	Normal	403 (96)	314 (99)	43 (80)	46(100)	
	Caesarian section	15 (4)	4 (1)	11 (20)	0	**<0.01**
Infant outcome						
	Normal	371 (89)	287 (90)	40 (74)	44 (96)	
	Still birth or neonatal death*	16 (4)	11 (4)	3 (6)	2 (4)	
	Needed special care	31 (7)	20 (6)	11 (20)	N/A	**<0.01**

APL, Above poverty line; BPL, below poverty line

*Still birth (n = 3)

*JSY facility was used as the reference group

The median age of the 418 mothers interviewed was 25 years. Half of the mothers belonged to BPL families (51%). More than a quarter (28%) of the women came from disadvantaged groups.

Ninety percent of the women had prior knowledge of the JSY program. The women learned about the program through the public health facility (40%), the village crèche worker (30%) or the ASHA (21%). Of the women who did not have prior knowledge, 70% (n = 31) delivered outside of the program i.e. private or at home.

The majority of deliveries (318/418; 76%) took place within the JSY program; 81% of all BPL mothers delivered in the program. Seventy percent of program deliveries occurred in a CHC and 26% in the district hospital. The main reasons reported for delivering in the program were because the facility was close to the home (44%), motivated by the JSY cash incentive (24%) or the perception that good services were available at the program facility (17%). All mothers that delivered in a JSY facility received the cash benefit; 86% received it within two weeks of delivering in a facility.

Of the 342 mothers that delivered in a facility (JSY or private), 37 were referred to another facility. Most referrals (n = 29) were to the district hospital from lower level public facilities. Eight mothers were referred to private facilities from the public. Of the 15 women who delivered by cesarean section, only four delivered in the program, accounting for 1% of all deliveries in the program. There was one maternal death reported in the study which occurred within the program.

### Who are the women not delivering within the JSY program? (Table 2)

**Table 2:** Final multivariate logistic regression analysis for factors determining place of delivery

	Adj OR (95%CI);	p value
**a) Delivery at a private hospital**		
Disadvantaged groups	0.45 (0.22-0.91)	0.03*
No formal education	0.51 (0.24-1.08)	0.08
Below poverty line	0.44 (0.22-0.89)	0.02*
Adequate ANC care	4.55 (2.09-9.91)	<0.01**
No knowledge about JSY	13.78 (5.23-36.28)	<0.01**
		
**b) Delivery at home**		
Disadvantaged groups	0.96 (0.43-2.15)	0.92
No formal education	2.61 (1.25-5.47)	0.01*
Below poverty line	0.81 (0.41-1.59)	0.53
No knowledge about JSY	3.00 (1.21-7.25)	0.02*
≥2 previous deliveries	11.68 (4.77-28.63)	<0.01**

Adjusted for type of caste, education and poverty level

*Significant

** *Highly Significant*

#### Private facility

After adjusting for caste, education and poverty status, mothers with no prior JSY knowledge were significantly more likely to deliver in one of the private facilities located in the study area. Also, women above the poverty line or those belonging to a general caste were more likely to deliver in a private facility. It was also associated with having a higher number of ANC visits. Education was not a significant predictor of delivery in a private facility. The main reasons reported for selecting the private facility were because women received ANC examinations there (29%), they believed it had a good reputation (21%) or because they were familiar and comfortable with the medical staff (19%).

#### At home

In the same multivariate model, women who were uneducated, multiparous or lacked prior knowledge of the JSY program were more likely to deliver at home. Deliveries at home were performed by traditional birth attendants. Poverty was not significantly associated with delivering at home. The main reasons for delivering at home were non availability of transportation (65%) or that the mother felt previous deliveries were 'easy' and so there was no need (26%).

### Role of the ASHA

The ASHA visited 86% of all mothers at least once during their pregnancy; 77% of mothers visited by the ASHA delivered within the program. However, only a minority of mothers received support from the ASHA in deciding the place of delivery (17%) or arranging transportation (13%), as these decisions were reported to be taken by husbands or other household members. Less than half (49%) of the women were accompanied by the ASHA to the hospital. Only 4% of mothers received a post-delivery home visit from the ASHA.

## Discussion

Demand-side financing has become increasingly popular in South Asia to help reduce access barriers for maternal healthcare and encourage antenatal care, skilled birth attendance or institutional delivery [20-23]. The programs intend to (i) reduce barriers to access of care, (ii) increase the use of maternal services i.e. institutional deliveries, and (iii) enhance the quality of care received through competition between providers (when the private sector is involved) [24]. India has implemented one of the largest conditional cash transfer programs, JSY, in the world for maternal health. This study, among villages where the cash transfer operates, explores program uptake and reasons for non-participation.

**Figure 2:**
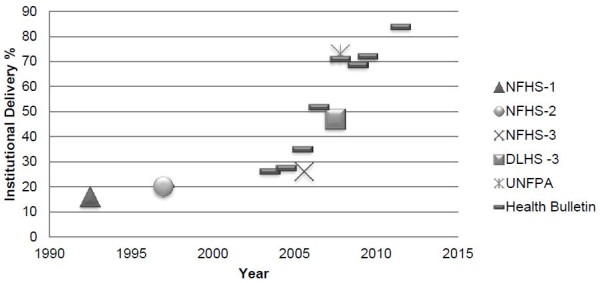
**Institutional Deliveries in Madhya Pradesh from 1992 to 2011**.

A number of national surveys and provincial data sources have demonstrated a steep increase in institutional deliveries both nationally and in Madhya Pradesh since the inception of the JSY program (figure 2) [15,19,25-28]. Lim et al. reported from an analysis of secondary data that the JSY program resulted in a 43.5% increase of institutional deliveries [7]. They reported a high uptake of JSY in Madhya Pradesh (44%). In our study, 89% of the women had an institutional delivery. While more women are giving birth in facilities, what remains unknown is if the increase in institutional deliveries has occurred among the most vulnerable groups. Demand-side financing programs have received criticism for not being able to reach the most disadvantaged women [9,10,12,29]. In this study, a high proportion of BPL mothers delivered within the program. Lim et al. also found that poor women were receiving the JSY cash transfer [7]. Conversely, a study from Powell-Jackson et al. in Nepal [6] reported some obstacles in reaching poor women. The program did not specifically target the poor, thus the distribution of the CCT was skewed towards the wealthier families that were more likely to have an institutional delivery. Nevertheless, within our study, there is still a subset of mothers delivering at home, who do not or cannot access emergency obstetric care under the program and remain at risk of maternal death.

The women giving birth in the program and at home had a similar socio-demographic background in terms of age, poverty level and caste; however they differed significantly with regard to education, parity, ANC, and awareness of the JSY program. In this study, the majority of women knew of the program through the existing health care facility. Poorly educated, multiparous women who did not receive adequate ANC were less likely to seek maternal health care services [7,30,31], hence were less likely to know about the program and also less likely to deliver within the program.

The women delivering at home reported two main reasons for not utilizing a facility for delivery; (i) difficulty accessing transportation and (ii) previous experience with 'easy' uncomplicated births. Janani Express Yojana ('Maternal Express Program') is a recent initiative (not in place at the time of our study), which addresses the first issue. Janani Express Yojana, a free ambulance service that transports pregnant women to a public facility for delivery, has to a large extent possibly remedied the transportation constraint [32]. The geographic areas that Janani Express Yojana covers are expanding, in the meanwhile adequate access to transportation continues to be a barrier for some.

The role and impact of the ASHA on program uptake has been debated in the literature [33,34]. While the ASHA is one of the critical community workers identified by the National Rural Health Mission (NRHM) to advocate the benefits of the program and support uptake, in our study her actions and influence were somewhat limited. The NRHM states the ASHA is responsible for identifying all the pregnant women in her village, assisting them with obtaining ANC, educating them about JSY benefits, arranging for transportation to the facility, accompanying the pregnant women to the facility for delivery, and promoting post natal care for the mother and child [13]. A large majority of the mothers were contacted at least once by the ASHA during their pregnancy. However, the ASHA did not play a significant role in informing the women about the program, arranging transportation or providing post natal care. While the ASHA probably had some influence on the increase in institutional delivery, it is evident that her ability to influence the mother's decision on where to deliver is limited as this decision is made by other household members. Therefore, to what degree she influences the uptake of the JSY program is uncertain. In order to promote universal coverage of women under the program, it is important to understand the real impact and influence of the ASHA on women's decision-making.

The socio-demographic profile of the women giving birth in private facilities was significantly different from the home birth group with regards to poverty status, education, caste, parity, and ANC receipt. The only similar characteristic shared was the lack of JSY program knowledge. The extreme diversity between the two groups was the reason for not combining them into a single non-program group during analysis.

It could be assumed that the women delivering in the private sector are not a priority target for the program. This stems from the assumption that women giving birth in private facilities can afford the expense and do not require the JSY benefit. More than half (52%) of the women who gave birth in a private facility were from a disadvantaged group. Of these 28 women, 21 reported they took a loan to cover their delivery expenditure at the private facility. The extent to which these loans influence these families' other expenditures are unknown.

There were a small number of referrals and caesarean sections recorded in the study. The referrals tended to move from lower health centers to the district hospital and from public to private facilities. Most likely there were some private to public referrals, however the small size of the study did not allow for this to be captured. The private sector performed a large majority of the caesarean sections. This may have been due to the direction of the referrals and that the most common delivery facility (CHCs) in the study area offered only basic emergency obstetric care.

### Program Processes

In this study, all women who participated in the JSY program received the cash benefit, 57% at the actual time of discharge and a further 28% within two weeks of delivery, implying a relatively well functioning program process. The success of a cash transfer program relies on the intended beneficiary receiving the incentive in a timely manner, otherwise the program risks falling into disrepute. Reports from other demand-side financing programs in Nepal [10] and Bangladesh [8] indicate the facilitation of the cash benefit or voucher funds was problematic due to lack of funds and poor procedural implementation of the scheme. However in our study, this does not appear to be a problem with JSY.

## Methodological Considerations

Many mothers (43%) that gave birth in 2009 were away from their homes at the time of the interview. The main reasons cited for not being at home included working outside the household and visiting their families in another village. This could have led to a selection bias as mothers working outside the house were likely to have been from poorer families, which could have influenced their chosen place of delivery. The study was also limited by the small sample size; particularly the small number in the non-program groups. Previous reports have indicated a relatively high uptake of the program in Madhya Pradesh. Ujjain is one of the better performing districts in regards to health within the province so the results may not be generalizable to other areas.

## Conclusions

There was significant program uptake in our study area with a large majority of poor women participating in the program. Proximity to the facility and the cash benefit were the two main reasons women participated in the program. Women who were uneducated, multiparious or lacked prior knowledge of the JSY program were more likely to deliver at home. These women reported difficulties accessing transportation. Although some barriers to the uptake of the program have been rectified, others still persist. The ASHA's influence on where the mother delivered appeared limited, potentially restricting her impact on the overall program uptake. There is a need to include these identified women in the program and an opportunity for program implementers to target them with new innovative strategies.

## List of abbreviations

ANC: antenatal care; ANM: auxiliary nurse midwife; APL: above poverty line; ASHA: accredited social health activist; BPL: below poverty line; CHC: community health center; CCT: conditional cash transfer; JSY: Janani Suraksha Yojana; NRHM: National Rural Health Mission; PHC: primary health center

## Competing interests

The authors declare that they have no competing interests.

## Authors' contributions

KS was responsible for research tool development, data acquisition and management, drafting the manuscript and assisted in data analysis. VD was responsible for conceptualizing the research question, contributed to study design, data management, and assisted with drafting the manuscript. ZEK was primarily responsible for data analysis. ADC contributed to conceptualizing the research question, study design, data analysis and drafting the manuscript. All authors contributed and approved the final manuscript.
